# Advanced therapy to cure diabetes: mission impossible is now possible?

**DOI:** 10.3389/fcell.2024.1484859

**Published:** 2024-11-19

**Authors:** Rokhsareh Rohban, Christina P. Martins, Farzad Esni

**Affiliations:** ^1^ Department of Internal Medicine, Division of Hematology, Medical University of Graz, Graz, Austria; ^2^ Department of Surgery, Division of Pediatric General and Thoracic Surgery, Children’s Hospital of Pittsburgh, University of Pittsburgh Medical Center, Pittsburgh, PA, United States; ^3^ Department of Developmental Biology, University of Pittsburgh, Pittsburgh, PA, United States; ^4^ UPMC Hillman Cancer Center, Pittsburgh, PA, United States; ^5^ McGowan Institute for regenerative Medicine, University of Pittsburgh, Pittsburgh, PA, United States

**Keywords:** diabetes, insulin-producing cells (IPCs), stem cells, cell therapy (CT), gene therapy (GT)

## Abstract

Cell and Gene therapy are referred to as advanced therapies that represent overlapping fields of regenerative medicine. They have similar therapeutic goals such as to modify cellular identity, improve cell function, or fight a disease. These two therapeutic avenues, however, possess major differences. While cell therapy involves introduction of new cells, gene therapy entails introduction or modification of genes. Furthermore, the aim of cell therapy is often to replace, or repair damaged tissue, whereas gene therapy is used typically as a preventive approach. Diabetes mellitus severely affects the quality of life of afflicted individuals and has various side effects including cardiovascular, ophthalmic disorders, and neuropathy while putting enormous economic pressure on both the healthcare system and the patient. In recent years, great effort has been made to develop cutting-edge therapeutic interventions for diabetes treatment, among which cell and gene therapies stand out. This review aims to highlight various cell- and gene-based therapeutic approaches leading to the generation of new insulin-producing cells as a topmost “panacea” for treating diabetes, while deliberately avoiding a detailed molecular description of these approaches. By doing so, we aim to target readers who are new to the field and wish to get a broad helicopter overview of the historical and current trends of cell- and gene-based approaches in β-cell regeneration.

## 1 Introduction

For many decades, the idea of injecting living cells or manipulating genes to treat various diseases was considered science fiction. Cell therapy originated in the 19th century, when Charles-Édouard Brown-Séquard (1817–1894) hypothesized that cells would have an increased life cycle when co-cultured with animal testicle debris (Charles-Édouard Brown-Séquard, 2006). Later in 1931, Paul Niehans (1882–1971) further assessed this hypothesis by injecting calf embryonic cell debris into a cancer patient for treatment purposes (Schengrund and Repman, 1979).

**TABLE 1:** illustrates a representative of registered clinical trials involving MSPCs in treatment of Diabetes (www.clinicaltrials.org *)*.

	Clinical Trial	Trial Status	Targeted Disease	Trial Phase	Powered by
1	Cellular therapy for Type 1 Diabetes using Mesenchymal Stem Cells (MSCs)	Recruiting Candidates/ Patients	Type I Diabetes Mellitus	Phase I	Medical University of Charleston, South Carolina, USA
2	BM-MNC & UC-MSC for Type II Diabetes Mellitus patients	recruiting candidates/patients	Type II Diabetes Mellitus	Phase I/II	Medical University of Indonesia Jakarta Pusat Jakarta, Indonesia
3	PROCHYMAL® (Human adult stem cells) for the treatment of recently diagnosed Type I Diabetes Mellitus (Type I DM)	completed	Type I Diabetes Mellitus	Phase II	University of Alabama & Stanford University, USA
4	Mesenchymal Stem Cells (MSCs) to treat Type II Diabetes	active, not recruiting	Type II Diabetes Mellitus	Phase II	Chinese PLA General Hospital Beijing, China
5	Outcomes of expanded autologous BM-derived MSC therapy in Type II Diabetes	completed	Type II Diabetes Mellitus	Phase I/II	Vinmec Research Institute of stem Cell & Gene Technology, Hanoi, Vietnam

Gene therapy is the therapeutic delivery of nucleic acids into a patient’s body to treat a disease. The possibility to directly change human genes for therapeutic purposes was first introduced around half a century ago (Wirth et al., 2013), when Martin Cline made the first attempt to modify human DNA (Wirth et al., 2013). In 1989 the first gene transfer in humans was successfully performed and the National Institutes of Health (NIH) approved the procedure (Maria et al., 1997). It was only in 1990 that direct human DNA insertion into the host nuclear genome was carried out by Anderson and colleagues (Vandebroek and Schrijvers, 2007). The aim was to set up a gene therapy strategy to treat genetic malignancies or find an ultimate cure for them (Vandebroek and Schrijvers, 2007). Since then, scientists have studied the biological mechanisms of numerous human hereditary and physiological diseases. They have discovered new paths leading to ground-breaking cell- and gene-based therapeutic interventions (Kitada et al., 2018). Progress in modern medicine has resulted in novel and potentially effective treatment options through advances in cell- and gene therapy (Anguela and High, 2019; Zakrzewski et al., 2019).

These all indicate that advanced medicine that was once thought of as a medical fantasy has now transformed into a break-through that reshapes the potential for novel therapeutics, in conjunction with other cutting-edge technologies.

## 2 β-cell regeneration

Neogenesis and proliferation have been considered as two major mechanisms leading to tissue and organ regeneration (Forbes and Rosenthal, 2014). Neogenesis is generating new cells from other cell types. It can be done either through differentiation of undifferentiated cells to achieve a specific cell fate, or conversion of one terminally differentiated cell type to another cell type (Demeterco et al., 2009; Kaslin et al., 2008). Proliferation, on the other hand, relies on the expansion of pre-existing cells. Stem-, multipotent-, or progenitor cells are considered major key players in neogenesis that would promote cell therapy (Kaslin et al., 2008; Zhou and Melton, 2018). They can be used for therapy either untreated or treated with viral vectors, chemical druggable small molecules, siRNA, etc. (Zhi et al., 2016; Wegman et al., 2013; Pavathuparambil Abdul Manaph et al., 2019). Genomic-altered stem cells have been already used to correct the mutations causing diseases (Daley and Scadden, 2008; Zou et al., 2011; Naldini, 2011). Genetic manipulation of the stem- and progenitor cells have been also used as a strategy to make these cells compatible for delivery of the desired therapy to target cells, tissues, or organs (Gonçalves and Paiva, 2017). These therapeutic strategies usually work upon cell transplantation or promoting proliferation of healthy cells.

In the pancreas, the endocrine cells residing within the islets of Langerhans are responsible for maintaining blood glucose levels. Glucose-responsive, insulin-producing cells in the islets are referred to as “β-cells“ and are crucial in maintaining this balance (Zhou and Melton, 2018; Cade and Hanison, 2017; Jennings et al., 2015). When β-cell depletion overtakes β-cell generation, the overall number of insulin-producing cells decreases, and a shortage of insulin becomes evident. A significant reduction in the number of functional β-cells, either through cellular loss or dysfunction contributes to the incidence of diabetes mellitus. Thus, developing regenerative strategies for β-cell replacement either through neogenesis or enhanced proliferation has been a key focus area of diabetes research. Cell and gene therapy pro β-cell regeneration aims to develop strategies for the treatment and a cure of diabetes through regenerative approaches. The wide range of regenerative strategies involve enhancing β-cell proliferation, or β-cell neogenesis (Pavathuparambil Abdul Manaph et al., 2019; Nir et al., 2007; Aguayo-Mazzucato and Bonner-Weir, 2018; Butler et al., 2007; Wang et al., 2015; Vetere and Wagner, 2012; Granger and Kushner, 2009). The latter entails either differentiation of embryonic or adult stem cells, or alternatively trans-differentiation of terminally differentiated cells into newly formed insulin-producing β-like-cells (Demeterco et al., 2009; Zhou and Melton, 2018; Pavathuparambil Abdul Manaph et al., 2019; World Health Organization, 2016). Notably, a successful cure for diabetes should also entail strategies to protect these newly formed β-cells, especially in an autoimmune setting (Halban et al., 2010; Chen et al., 2017; Bonora, 2008; Infante et al., 2019).

**FIGURE 1:**
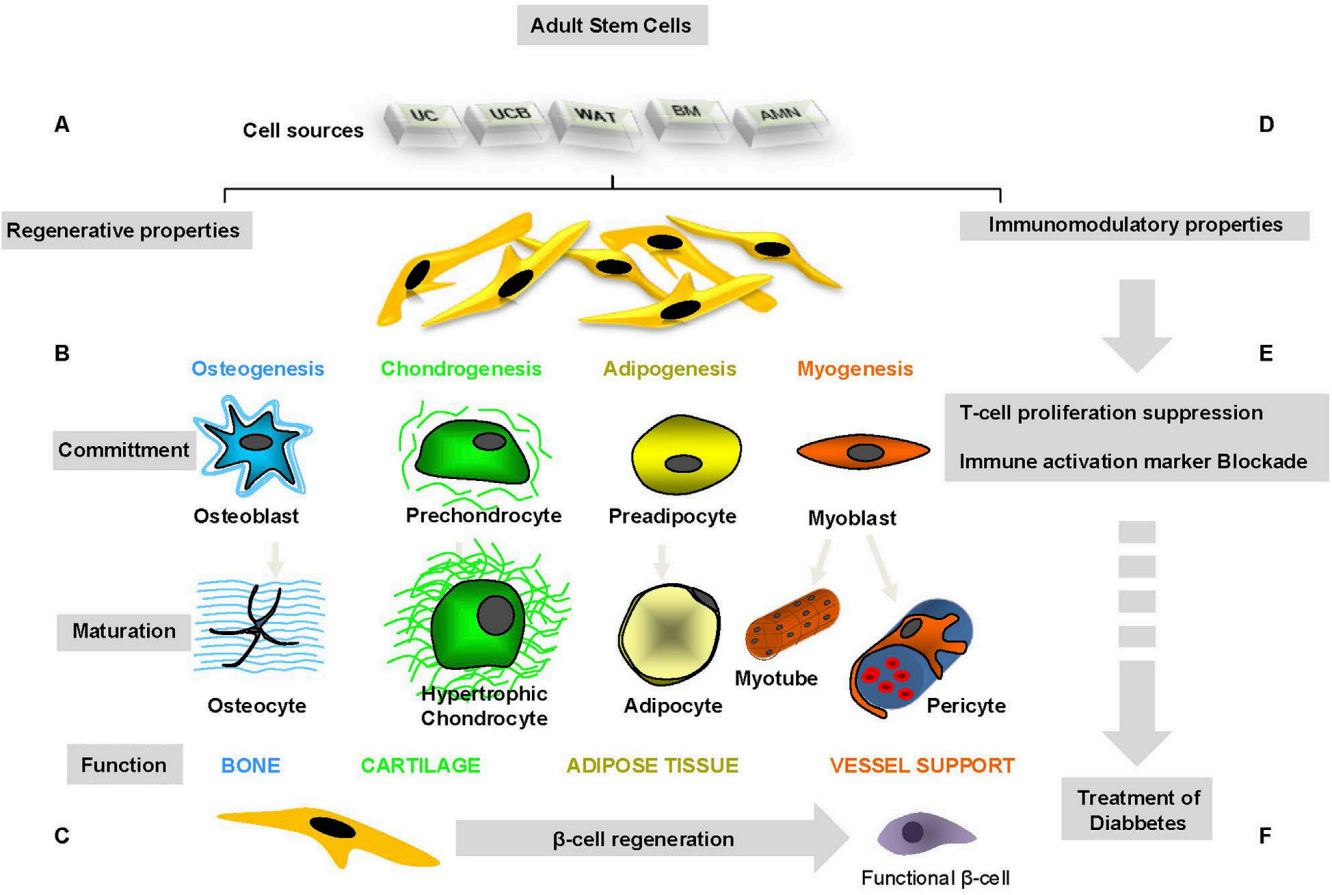
Regenerative and immunosuppressive capacity of tissue-specific stem cells.

Figure 1 illustrates regenerative and immunosuppressive capacity of tissue-specific stem cells.

### 2.1 β-cell proliferation

β-cell proliferation is most prominent during embryogenesis and the subsequent postnatal growth. Following this initial burst, the proliferative capacity of β-Cells is maintained at a low ratio throughout adulthood (Dahiya et al., 2024; Granger and Kushner, 2009; Jacovetti and Regazzi, 2022; Spears et al., 2021).

Studying the mechanisms that control β-cell proliferation with the overall goal of generating new endogenous β-cells has been a main focus for diabetes therapy research. Although the “proliferation” approach could potentially compensate for the reduced number of functional β-cells in Type 2 Diabetes, the success of such a strategy for treating Type 1 Diabetes is rather obscured, as reoccurrence of autoimmunity against new β-cells remains a concern. β-Cell proliferation strategies may open a door to combination therapy in diabetes treatment (Eguchi et al., 2022b; Xie et al., 2023). Combination therapies of proliferation agents with immunosuppressant and anti-oxidative substances have been shown to improve overall long-term outcomes of diabetes treatment. These therapeutic approaches have been highlighted in numerous reports (Eguchi et al., 2022a; Ludvigsson, 2016).

Given that β-cell proliferation in both human and rodent islets has been extensively reviewed elsewhere (Bernal-Mizrachi et al., 2014; Stewart et al., 2015; Elghazi et al., 2007; Rafacho et al., 2009; Elena et al., 2021; Cui et al., 2024), here we will primarily focus on recent advances in β-cell neogenesis.

### 2.2 β-cell neogenesis

It has long been presumed that the longevity of adult β-cells primarily relies on their replication capacity rather than neogenesis. Nevertheless, ongoing research tries to explain whether this doctrine holds for humans as well (Dor et al., 2004). The generation of insulin-producing cells to compensate for their absolute or relative shortage in type 1 and type 2 diabetes is an obvious therapeutic strategy. The low proliferative rate of pancreatic β-cells has led to the search for other sources of β-cell generation such as embryonic or adult stem cells, progenitor cells, facultative stem cells or terminally differentiated cells residing within or outside the adult pancreas. In this section, we will highlight some of these efforts (Márquez-Aguirre et al., 2015; De Haro-Hernández et al., 2004; Ye et al., 2016; Granger and Kushner, 2009).

To address the β-cell defective characteristic in T1 and T2 diabetes, researchers have focused on generating pancreatic β-cells from stem cells. They have also concentrated on re-building the β-cells' normal cellular niche (Choi et al., 2004; Zhang et al., 2013; Santana et al., 2006; Roche et al., 2009). Zhang et al. studied the functions of pancreatic islets isolated from human fetal pancreatic progenitor cells *in vitro* and *in vivo*. In their study, human fetal pancreatic progenitor cells were expanded in a culture medium enriched with fibroblast growth factor (FGF) and leukemia inhibitor factor (LIF). In order to direct the cells towards pancreatic endocrine cell differentiation, glucagon-like peptide-1 (GLP-1) and Activin-A were added to the cell culture medium. α- and β-cell endocrine and exocrine functions were analyzed by immunofluorescent staining and ELISA technique. Islet-like structures were transplanted into renal capsules of diabetic nude mice to evaluate the functions of these islets *in vivo*. Immunohistochemistry staining for human C-peptide and human mitochondrion antigen was used to show the human origin of the cells and viability of grafted islets (Zhang et al., 2013). The differentiated cells expressed insulin, glucagon, glucose transporter-1 and -2 (GLUT1 and GLUT2) and voltage-dependent calcium channel (VDCC) (Zhang et al., 2013). These cells were also able to form islet-like structures containing α- and β-cells. These islet-like structures were glucose responsive, leading to normoglycemia in diabetic nude rodents post transplantation (Zhang et al., 2013).

## 3 Gene therapy for β-cell regeneration

Gene therapy involves delivering molecular and cellular components that are known to promote β-cell growth or regeneration. Scientists have already developed β-cell and tissue-specific gene delivery vector systems. These systems should allow for studying the therapeutic impact of inhibition and/or overexpression of cellular factors required for β-cell regeneration (Ortis et al., 2010). A study has demonstrated the impact of TGF-β1 gene therapy in preventing islets from destruction due to autoimmunity. The method has also been shown to promote islet regeneration, leading to diabetes cure in diabetic NOD mice (Luo et al., 2005).

Another study showed that streptozotocin-induced diabetes in rats is reversed by betacellulin and pancreatic duodenal homeobox-1 treatment using gene therapy (Chen et al., 2007). In a different research by Ikeda et al., the impact of several low-dose STZ treatments and pancreatic Reg3b–Glp-1 gene therapy on the gene expression profiles of pancreatic islets have been studied. Their results unraveled induction of p53-responsive genes and suppression of a numerous diabetes-related genes upon treatment with STZ. Overexpression of REG3B–GLP-1 in the pancreas protected β-cells from destruction upon STZ treatment and inhibited hyperglycemia in mice (Tonne et al., 2013).

Although gene therapy for Diabetes treatment has not been studied as intensively as cell therapy possibly due to potential ethical issues and safety challenges, this approach seems promising for finding a feasible cure for diabetes mellitus.

## 4 Stem cells in β–cell generation

### 4.1 Embryonic stem cells (ESCs)

ESCs have demonstrated promising potential to differentiate into various committed cell types, including pancreatic β-cells. The self-renewal capacity of ESCs makes them a competent source for insulin-producing β-like cells.

Other reports have indicated that pancreatic endocrine progenitor cells from ESCs can give rise to insulin-producing cells when transplanted into mouse models (Bruin et al., 2013; Liu and Lee, 2012).

Bruin et al. had previously demonstrated that human embryonic stem cell (hESC)-derived pancreatic progenitor cells can be differentiated into insuline producing cells when transplanted under the kidney capsule in a mouse model of diabetes (Bruin et al., 2013). To overcome the undesired diffrentiation of hESC into bone and cartilage cells, they developped an improved differentiation protocol that aimed to prevent the formation of off-target mesoderm tissue post-transplantation. They also reported that the variation within the host tissue environment has an impact on the development of pancreatic progenitor cells *in vivo* (Bruin et al., 2013).

Also, Liu et al. presented an efficacy strategy for the differentiation of mouse embryonic stem cells into insulin-producing cells using a two-step differentiation protocol for the formation of endoderm in monolayer cellular culture by activin A, and the differentiation of this monolayer endoderm into insuline producing cells using substances such as nicotinamide, insulin, and laminin. They have demonstared that the differentiation process is successfully obtained with appx. 7 days and the newly formed insuline producing cells are capable of realising insulin in a dose dependent manner based on the amount of added glucose (Liu and Lee, 2012). Therefore, these cells demonstrated an appropriate response to changes in serum glucose levels (Liu and Lee, 2012).

Further, in attempts to use differentiation protocol for neuronal progenitor cells, a few numbers of nestin^+^ ESCs were shown to differentiate into β-like cells. However, a handful number of cells within the small cell population expressed specific insulin genes. These cells were also incapable of Pdx-1 expression (Memon and Abdelalim, 2020; Hori et al., 2005; Ben-Yehudah et al., 2009; Pan et al., 2019)^,^ inspite of possesing neuronal cell phenotype (Pan et al., 2019).

The study showed that the cells continued differentiation after transplantation *in vivo*, leading to insulin secretion in mice. This result has been also in accordance with other reports indicating that human ESCs can respond to environmental signals, and differentiate further according to external signals (Vazin and Freed, 2010; Yim and Sheetz, 2012; Mullen and Wrana, 2017; Mossahebi-Mohammadi et al., 2020; Lanner and Rossant, 2010; Huang et al., 2020). It is to conclude that in this case, the message for ESCs has been to further differentiate to endocrine pancreatic islet cells (Huang et al., 2020; Jacobson and Tzanakakis, 2017). Some studies also investigated and compared the murine and human-derived cells *in vivo* (Yu and Xu, 2020; Baeyens et al., 2018). They revealed that the islet development machinery in rodents (mouse) and human is conserved (Yu and Xu, 2020; Baeyens et al., 2018; Nair and Hebrok, 2015). In this regard, a supportive finding revealed that insulin-producing cells co-cultured with islet cells for approximately 1 month responds to glucose concentration under physiological condition (Oh et al., 2015; Campbell and Newgard, 2021). This study found a co-culture strategy that better promoted differentiation efficiency compared to chemical enforcement methods with soluble chemical factors (Pavathuparambil Abdul Manaph et al., 2019). Due to a limited knowledge around the mechanisms of cell maturation, this effective strategy for promoting islet maturity and regeneration has not yet been used in the clinical trials.

By now, scientists have been able to produce functional insulin-producing, glucose responsive β-like cells. However, the low efficiency of the presented methods and inability to regenerate highly competent β-like cells have been challenging. More recently, scientists have put a great effort in the improvement of the existing protocols for ESC differentiation into pancreatic progenitor cells (Ben-Yehudah et al., 2009; Mayhew and Wells, 2010; Shahjalal HM. et al., 2018; Memon et al., 2018; Wesolowska-Andersen et al., 2020). It should however be noted that the ethical and safety issues might diminish the efficacy of such treatment strategies. In this regard, the tumourigenicity of ESCs is one major concern and obstacle for the success of the therapeutic strategy. This appears as an important aspect for the patient’s health (Herberts et al., 2011). Advanced techniques in enhancing cell maturation and purification can ensure the safety procedure of implantation of fully differentiated, mature cells. These techniques involve fluorescence- and magnetic activated cell sorting, genetic selection, use of biomarkers, and embryonic cell lines. As a result, the risk of tumorigenesis can be reduced (Martin, 2017; Wuputra et al., 2020). The biggest problem, however, remains the ethical conflict about the isolation and harvest of human ESCs Therefore, the majority of studies have focused on animal models, which is not always translatable to human cell and organ machinery.

To date, a handful number of clinical trials for stem cell-based treatment of T1 diabets using human embryonic stem cells (hESC) have been registered (Park et al., 2024).

Notably, Schulz et al. created a product consisting of pancreatic endoderm cells from hESCs and a drug delivery system, (Schulz, 2015). This product went through phase 1/2 clinical trial. However, the study was terminated due to the lack of immunosuppressing strategy resulting in a host reaction against the implant (Pullen, 2018). On a second attempt, a Phase 1/2 clinical study involving 17 T1D patients was carried out using the modified product. This study showed successful engraftment and insulin secteration in significant number of the cases. Half a year post-implantation, significant number of the participants showed positive levels of C-peptide, demonstrating the potential of the product as a promissing method for T1DM treatment (Shapiro et al., 2021). The results of this clinical study underscores innovative approach that utilizes pancreatic endodermal cells derived from hESC for treating diabetes. The hESCs have been also genetically modified by the CRISPR/Cas9 technology. This modification also resulted in enhancement of cellular survival rate against the patient’s immune system, deminishing the risk of graft *versus* host disease (Ellis et al., 2023). In addition, another product consisting of differentiated pancreatic islet cells derived from human for treating T1D has been desigened for clinical investigations (Reichman et al., 2023). As of yet, the data analysis from this study has revealed promissing results, demonstrating restored insulin production in the first couple of participants in the study.

With all being discussed about the impact of embryonic stem cells in generating insuline producing cells, however, the ethical and safety concerns exist remain, hampering the potential of using these cells in clinical trials. Besides, the cell differentiation process itself may cause partially undesired complications in the cell phenotype that might lead to immunogenicity and force an unforeseen immune reaction in the body. Here, therapies that protect new β-cells from being destructed are critical. Hence, the β-cell protection side approach for applying cell therapy strategies pro β-like cell regeneration comes in handy. The immunomodulatory therapy in which mesenchymal stem cells can be also involved promotes the development of efficient protocols for the production of ESC-derived pancreatic β-cells. several protocols have been developed for differentiation of human ESCs towards insulin-producing cells (Naujok et al., 2011; Jiang et al., 2007; Trounson, 2007; Lee and Chung, 2011; Nagaya et al., 2019; White et al., 2008; Mastracci and Sussel, 2012).

### 4.2 Adult stem cells

Adult stem cells are the most viable source for cell therapy in several disease models (Bai, 2020; Editorial; Mousaei Ghasroldasht et al., 2022; Sng and Thomas, 2012). They do not demonstrate ethical issues in most countries and seem to provide an unlimited resource for advanced regenerative therapies. Many reports indicated that β-like insulin-producing cells can be generated from stem and progenitor cells residing in bone marrow, adipose tissue, liver, spleen, neuronal tissues, and umbilical cord blood. Precise and detailed studies are required to identify the most effective tissue-specific adult stem cells for generation of insulin-producing, glucose-responsive fully committed β-like cells (Naujok et al., 2011). It is also much needed to develop more efficient differentiation protocols to allow for testing these strategies in clinical studies.

Despite an obvious need for further assessments, optimizations, and clinical trials, the initial success of the (re)generation of glucose-responsive insulin-secreting β-like cells gives promise for a therapeutic strategy for diabetes using stem cell-based cell therapy (Pokrywczynska et al., 2013).

Stem and progenitor cells can be derived from a variety of tissues such as cord, blood, bone marrow, adipose tissue, oral cavity tissues such as pulpa, periost and spongiosa, and epithelium. These cells have been characterized, expanded and applied for transplantation procedures in which adult stem cells give rise to committed cells such as osteocytes, adipocytes, and chondrocytes as well as β-like insulin-producing cells (Choi et al., 2004; Rohban and Pieber, 2017).

Several scientific reports have indicated the role of tissue-specific adult mesenchymal stem and progenitor cells (MSPCs) in the regeneration of insulin-producing β-like cells. In most of the studies, MSPCs (Rendra et al., 2020) isolated from bone marrow, adipose tissue, and umbilical cord blood have been shown to contribute more effectively in the regeneration of β-like cells compared to cells from other tissue of origins (Rohban and Pieber, 2017; Jun HS. and Park EY., 2009; Liu D. et al., 2020).

**FIGURE 2:**
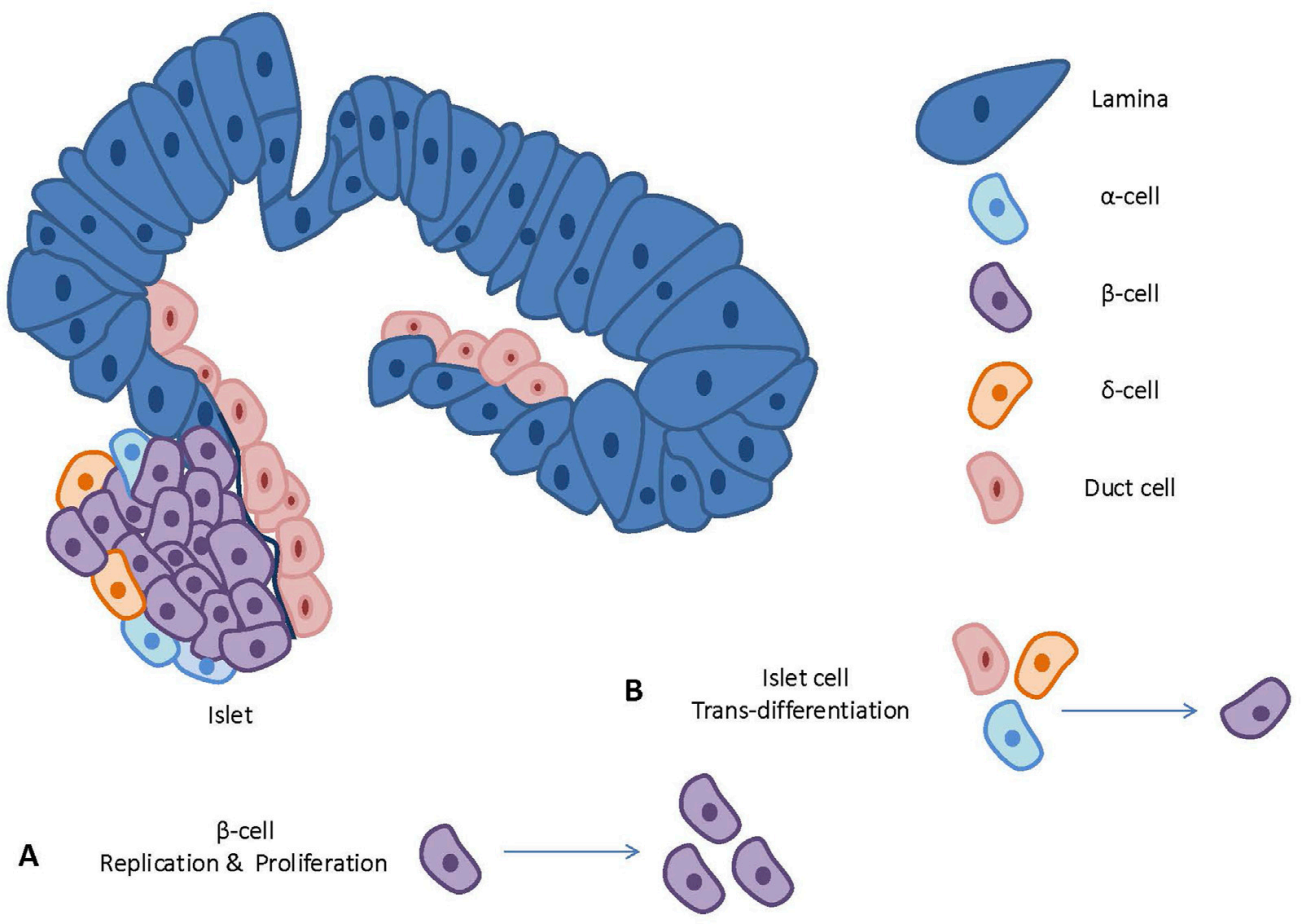
An overview on cellular playmakers and strategies toward β-cell regeneration.

Figure 2 depicts an overview on cellular playmakers and strategies toward β-cell regeneration.

### 4.3 Tissue-specific stem cells in β-cell regeneration

#### 4.3.1 Bone marrow-derived stem and progenitor cells (BM-MSPC)

While numerous studies have demonstrated the potential of BM-MSPC to differentiate into hematopoietic-, endothelial-, or mesenchymal lineages (Hassan and El-Sheemy, 2004; Vasanthan et al., 2020; Charbord, 2010; Ullah et al., 2015), there are contradictory reports on the ability of BM-MSPC in generating insulin-producing glucose-sensitive β-like cells. A study conducted by Ianus and colleagues showed that transplanted bone marrow cells gave rise to functional pancreatic β-like-cells without cellular fusion *in vivo* (Ianus et al., 2003). The study involved murine bone marrow cells from male donor animals expressing green fluorescent protein (EGFP). The transcription of the insulin gene was induced and the cells were transplanted into irradiated female mice as the recipients. The study showed that EGFP^+^/insulin^+^ cells in the pancreas of recipient mice appear at approximately 1-month post-transplantation (Ianus et al., 2003). However, some similar studies were not successful in reproducing their results (Lechner et al., 2004). Therefore, to date, there is no evidence showing that trans-differentiation of bone marrow cells into pancreatic β-cells in the mice is possible. Rather, it was concluded that the transplanted bone marrow cells produce components that promote the regeneration of β-like cells in the recipient animals. Moreover, since all these works used heterogeneous bone marrow cells, it is not completely clear which exact cells contributed to the regeneration of insulin-producing cells or endogenous pancreatic β-cells. These experiments have been also performed in mice models using murine progenitor cells for transplantation. The ability to recapitulate these findings using human samples remains unknown and deserves concrete investigations.

Bone marrow (BM) cells often consist of two different stem cell populations: hematopoietic stem cells (HSCs) and mesenchymal stem and progenitor cells (MSPCs). One study showed that mouse BM-derived MSPCs cells were able to undergo differentiation and they express pancreas-related biomarkers such as insulin I and II, Glut2, glucose kinase, islet amyloid polypeptide, nestin, pancreatic duodenal homeobox-1 [PDX-1], and Pax6 (Tang et al., 2004). Insulin and C-peptide production was also detected using immunocytochemistry and electron microscopy (Tang et al., 2004). Upon transplantation of these differentiated cells into streptozotocin (STZ)-induced diabetic mice models, hyperglycemia was altered and reversed after approximately 7 days (Tang et al., 2004).

Other studies have revealed that BM-derived MSPCs could trans-differentiate into insulin-producing cells using defined cell culture conditions (Dave, 2014; Gabr et al., 2017) enriched with extrinsic insulin-promoting factors and substances including activin A, nicotinamide, epidermal, hepatocyte, and fibroblast growth factors (Dave, 2014). They showed that transplantation of human BM-MSPCs into STZ-induced diabetic non-obese diabetic (NOD) mice elevated the number of endogenous murine β-cells. As a result, it led to increased insulin secretion, suggesting that MSPCs are likely to facilitate endogenous β-cell regeneration rather than contributing to neogenesis (Hashemian et al., 2015; Zang et al., 2017; Solis et al., 2019).

A number of reports suggest that BM-MSPCs can be converted into insulin-producing β-like cells by minimal differentiation factors such as nicotinamide (Rad et al., 2015) and exendin-4 for induction stages of differentiation (Moshtagh et al., 2013) or through gene therapy using genetic manipulation methods as dscribes by Efrat et al. (Karnieli et al., 2007). Briefly, in this study BM-MSC from 14 human donors were undergone Pdx1expression procedure and the differentiation of these cells toward the β-cell phenotype was examined. The study indicated that the cells failed to express NEUROD1, a crucial transcription factor in differentiated β-cells, Despite that, a significant insulin production, and also glucose-stimulated insulin production, were detected *in vitro* (Karnieli et al., 2007). According to this report, the cells have been then transplanted into STZ-diabetic immunodeficient mice for further differentiation which led to NEUROD1 induction and hyperglycemia reduction (Karnieli et al., 2007). Overexpression of Pdx-1 in human BM-MSPCs has been also recognized by others. This led to the differentiation of the cells into insulin-producing cells with β-like cell characteristics and their relevant biomarkers such as Pax-6 and Pdx-1 (Mimeault and Batra, 2008; Trivedi et al., 2010; Brovkina and Dashinimaev, 2020).

Human BM-MSPCs, when transfected with three transcription factors Pdx-1,NeuroD, and Ngn-3, have been shown to differentiate into insulin-producing cells *in vitro* (Chandra et al., 2009; Timper et al., 2006). Transplantation of these differentiated cells decreased blood glucose levels in diabetic mouse models (Pokrywczynska et al., 2013).

BM subpopulations treated with cytokines such as IL-3, IL-6, IL-11 *in vitro*, and then transplanted into irradiated mice has been shown to migrate to the pancreatic islets and convert to insulin-producing cells *in vivo* (Wada et al., 2019). The precise mechanism through which this procedure takes place is not fully known yet.

Although there are contradictory reports about the trans-differentiation potential of BM cells, stem cells residing in BM are considered viable options for the regeneration of insulin-producing β-cells.

#### 4.3.2 Adipose tissue-derived mesenchymal stem and progenitor cells (AT-MSPC)

Adult stem cells residing in human adipose tissue (thereafter referred to as Adipose tissue-derived stem cells (ADSCs)) can be isolated from the stromal vascular fraction of adipose tissue (AT) (Rohban and Pieber, 2017), and represent high population doubling capacity (Luna et al., 2014; Castro-Oropeza et al., 2020).

The procedure is relatively risk-free and isolated cells can be used for therapeutic purposes (Mastrolia et al., 2019; Chu et al., 2019). Human multipotent stromal cells isolated from the AT are likely to differentiate into adipogenic, chondrogenic, osteogenic, and myogenic cells and pericytes when seeded and maintained under specific conditions in differentiation culture medium (Rohban and Pieber, 2017; Rohban et al., 2017; Rohban R. et al., 2016; Rohban et al., 2013; Bunnell et al., 2008; Rohban Rokhsareh et al., 2016).

To date, only a handful of reports have been in favor of regenerative potential of ADSCs as a therapeutic opportunity to give rise to insulin-producing β-like cells. It has been already revealed that human ADSCs can differentiate into insulin-producing cells *in vitro* under specific differentiation medium conditions (Rad et al., 2015; Moshtagh et al., 2013): As of the pre-induction stage, low-glucose DMEM enriched with FBS), ß-mercaptoethanol, and nicotinamide has been used. In the induction stage, however, high-glucose, FBS-free DMEM, ß-mercaptoethanol, and nicotinamide have been added to the cell culture. Upon this two-stage differentiation protocol, the committed cells express pancreatic developmental biomarkers such as Isl-1, Pdx-1, and Ngn-3 as well as insulin, glucagon and somatostatin (Karnieli et al., 2007; Mimeault and Batra, 2008). However, a precise and detailed study on the functionality of these differentiated cells is still missing. This leads one to speculate that the regenerated β-like cells originated from AT stem cells might not be suitable candidates to run a promising clinical trial for restoring β-cell functionality. However, in recent years, several studies have been performed focusing on AT-MSPC in cell therapy. In some studies, mesenchymal stem and progenitor cells (MSPC) isolated from human adipose tissue were differentiated into insulin-producing cells. They cultured the MSPC for 3 days using the β-like cell differentiation protocol as described by Triverdi et al. (Trivedi et al., 2010). They then injected the differentiated cells together with bone marrow-derived hematopoietic stem cells into diabetic patients through infusion. The results revealed a 30 %–50% reduction in the exogenous insulin requirement. The C-peptide levels were also boosted 4- to 26-fold in the serum. The study suggested that transfusion of differentiated ADSCs might be a promising strategy for treatment of diabetes (Jun HS. and Park EY., 2009; Trivedi et al., 2010; Brovkina and Dashinimaev, 2020).

Chandra et al. also used the same 3-step differentiation protocol on murine ADSCs (Chandra et al., 2009) aiming to differentiate the cells into functional β-like cells (Jun HS. and Park EY., 2009; Chandra et al., 2009). They modified the protocol by converting mesodermal murine ADSCs to definitive endoderm, then pancreatic endoderm, and finally to pancreatic hormone-expressing cells. They showed that these cells were able to express endocrine biomarkers and produce C-peptides upon glucose level change. Further, they transplanted these differentiated islet-like aggregates i. p. into mature STZ-induced diabetic mice. Interestingly, the results revealed a restored state of normoglycemia 2 weeks post-transplantation (Chandra et al., 2009).

Other studies demonstrated the successful differentiation of functional β-like insulin-producing cells from stem cells isolated from human adipose tissue (Timper et al., 2006; Pokrywczynska et al., 2013; Wada et al., 2019; Enderami et al., 2018). In these studies, the cell isolation protocol was carried out using 2D (Wada et al., 2019) and 3D (Enderami et al., 2018) cell culture protocol. All in all, these studies reported that the Pdx-1^+^-transduced ADSCs derived from human or mouse tissue can give rise to insulin-producing β-like cells under specific culture differentiation conditions (Kajiyama et al., 2010). Together, these reports highlight ADSCs as potential source for cell therapy in diabetes.

#### 4.3.3 Umbilical cord blood-derived stem cells (UCB-MSPC)

Umbilical cord blood (UCB)-derived MSPCs is one of the promising stem cell sources in regenerative therapy (Han et al., 2019). These cells can be isolated with minimal complications, with no surgery or pain involved for the donor. UCB-MSPCs have already expressed multi-lineage differentiation potential under specific differentiation culture medium (Peters et al., 2010). Studies have reported that UCB stem cells can be differentiated into insulin-producing cells (Jun HS. and Park EY., 2009; Babiker et al., 2019; Van Pham et al., 2014).

UCB-derived embryonic stem cells expressing stage-specific antigen 4 (SSEA4) and octamer 4 (Oct4) biomarkers differentiate into insulin-producing β-like cells (Sun et al., 2007). They were capable of C-peptide protein production as insulin precursor and could express insulin (Sun et al., 2007). Denner et al. reported that human UCB-derived stem cells obtained can give rise to insulin-producing cells *in vitro* (Denner et al., 2007). Briefly, CD133^+^ CD34^+^ cells have been isolated from normal pregnant women post C-section delivery and expanded using a cytokine induced expansion protocol (Denner et al., 2007). The cells have been then subjected to an established protocol for differentiating mouse embryonic stem cells toward pancreatic phenotype by using a directed engineering method as described by Denner and colleagues (Denner et al., 2007) resulting in insulin producing cells *in vitro*.

Another study reported that the insulin-producing cells differentiated from UCB-derived MSPCs administered with extracellular matrix were able to produce insulin. However they failed to respond to glucose levels (Gao et al., 2008; Hu et al., 2009). Despite being insulin secreting competent, this missing potential in the regenerated β-like cells hampered the overall perspective of using UCB-MSPCs in cell therapy trials for diabetes and requires further investigation and optimization to realize the impact of UCB-MSPCs in generation of fully functional insulin-producing cells.

Yoshida et al. emphasized the regenerative potential of UCB-stem cells in giving rise to insulin-producing cells, when they infused UCB-stem cells into immunodeficient NOD. scid mouse models by intravenous (i.v.) injection (Yoshida et al., 2005). This report demonstrated an ability to detect UCB-derived insulin-positive cells in the pancreas of the recipient animals, suggesting that UCB-stem cells can commit into insulin-producing cells both *in vitro* and *in vivo*.

#### 4.3.4 Hepatic stem cells

Several studies have shown that human hepatocytes and liver cells can give rise to insulin-producing cells through directed expression of essential β-cell transcription factors such as Pancreas/duodenum homeobox protein 1 (PDX1), Neurogenin-3 (Ngn-3), *etc.* (Pan et al., 2019; Jun HS. and Park EY., 2009; Burns et al., 2004). In this regard, several manipulation strategies such as genetic manipulation, small molecule interventions and microenvironment modulation for bioengineering processes have been tested (Pavathuparambil Abdul Manaph et al., 2019; Kondo et al., 2017; Weir, 2004).

Notably, a differentiation protocol to change human induced pluripotent and human embryonic stem cells (hiPSC/hESC) to insulin-producing cells through small-molecule inducers has been established (Kondo et al., 2017). Chemical library screening has been performed to recognize small molecules that induce insulin production in hESC-derived pancreatic and duodenal homeobox 1 (PDX1)+pancreatic progenitor cells. Amongst different compounds, sodium cromoglicate (SCG) as a small molecule improved the formation of pancreatic endocrine cells from numerous hiPSC/hESC lines and mouse embryonic pancreatic explants. This small molecule could also promote differentiation of endocrine precursors, leading to generation of insulin-producing cells from hiPSCs/hESCs (Kondo et al., 2017).

Jin et al. have transduced immortalized liver epithelial progenitor cells with Pdx-1 biomarker in the presence of cytokines and growth factors (Jin et al., 2008). They were able to differentiate these cells into insulin-secreting cells that were also glucose responsive. They then transplanted the β-like cells into STZ-induced diabetic NOD. scid mouse models. Their study revealed that the transplantation of these cells in diabetic mouse models reduced blood glucose levels and ameliorated diabetes in these animals (Jin et al., 2008).

Pdx-1 in the liver of mice has also been shown to promote insulin production in STZ-induced diabetic animals (Cao et al., 2004). Moreover, the active form of PdX-1 (Pdx1-VP16) together with NeuroD or Ngn-3 was expressed in hepatocytes and facilitated the differentiation of hepatocytes into insulin-expressing cells (Yang, 2006; Ham et al., 2013).

In another work, oval stem cells isolated from the rat have successfully differentiated into hepatocytes (Shupe et al., 2009). These cells have also given rise to different pancreatic islet cells when they have been transferred to a glucose-enriched culture medium. Also, according to Yang et al., rat-driven hepatic oval stem cells can differentiate into hepatocytes and bile duct epithelium (Yang et al., 2002). These cells were also able to convert into pancreatic endocrine hormone-producing cells if cultured in a high-glucose medium (Yang et al., 2002). The cells secreted insulin in response to glucose, and showed the potential to reverse hyperglycemia in a diabetic NOD-scid mouse (Yang et al., 2002). Their findings indicate that primary adult liver stem cells undergo differentiation in a non-lineage-restricted pattern (Yang et al., 2002). Human liver progenitor cells expressing Pdx-1 have been also shown to trans-differentiate to insulin-producing cells as described by Lee and colleagues (Lee et al., 2021).

#### 4.3.5 Pancreatic stem and progenitor cells

Several studies have provided evidence for the existence of stem and progenitor cells in the adult pancreas: these cells have been detected close to pancreatic ducts and within the islets in human T1D patients and partially pancreatectomy model in rodents (Zhou and Melton, 2018; Bhartiya, 2016; Chen et al., 2020). It has been previously shown that islet-like aggregates formed from mouse pancreatic ducts and human pancreatic islets, are able to secrete insulin upon glucose stimulation and express islet specific biomarkers (Khatri et al., 2020). The expression of Ngn-3 in pancreatic ductal cells led them to trans-differentiate into insulin-expressing cells (Gomez et al., 2015). Also, the treatment of human islets containing both ductal and acinar cells with a combination of epidermal growth factors and gastrin molecules directed these cells toward neogenesis of β-cells. At the same time, it accelerated the active and functional β-cell population within the islets (Juhl et al., 2010). In a report published in 2020, Wang et al. described an unidentified protein C receptor+ (Procr+) cell population in murine pancreas using single-cell RNA sequencing (scRNA-seq) (Wang et al., 2020). The cells homing to islets could not express differentiation markers while expressing epithelial-to-mesenchymal transition characteristics. Genetic lineage tracing technology revealed that Procr + islet cells expand and give rise to endocrine cell types (Wang et al., 2020). Cell sorting technology also revealed that Procr + cells (approx. ∼1% of islet cells) can give rise to islet-like organoids *in vitro*. The organoids were able to respond to different glucose- and insulin levels. After being transplanted in diabetic mice, these organoids could reverse disease (Wang et al., 2020). These findings indicated that a population of Procr + endocrine progenitors is present in the adult murine pancreatic islet (Wang et al., 2020) with the potential for being used in Diabetes therapy.

Hao et al. reported that human non-endocrine pancreatic epithelial cells co-transplanted with human fetal pancreatic tissue could result in endocrine cell differentiation in immune-compromised mice (Loomans et al., 2018). They speculated that the fetal pancreatic cells provided supporter factors and molecules that promote survival and differentiation of the epithelial cells. Another study reported that Ngn-3 expressing progenitor cells exist in the ducts of the adult mouse pancreas (Xu et al., 2008). In this study, the Ngn3^+^ cells isolated from the adult mouse pancreas have been shown to differentiate into glucose-responsive insulin-secreting β-cells (Xu et al., 2008).

Experiments carried out in adult pancreas indicated replenishment of β-cells and restoration of glycemic level in STZ-treated rodents (Corritore et al., 2016). This was due to trans-differentiation of ductal cells into insulin-producing cells upon treatment with growth factors or small molecules as described (Corritore et al., 2016). Reports suggested that β-cells in the pancreatic islets can be de-differentiated, re-differentiated or expanded through epithelial-mesenchymal transition (EMT) (Russ et al., 2009; Efrat, 2016). Interestingly, amylase^+^/elastase^+^ acinar cells were also able to convert into insulin-expressing cells (Okuno et al., 2007). Also, pancreatic ɑ-cell line induced by Pdx-1 was shown to secrete insulin in the presence of β-cellulin (Thowfeequ et al., 2007). These findings demonstrate that stem and progenitor cells residing in the pancreas, along with the cells mentioned above might be the origin of new islet cells and in particular insulin producing cells. The specific biomarkers that play a role in this process require further investigations.

## 5 Induced pluripotent stem cells (iPSCs)

Pluripotent stem cells can be made directly from a somatic cell. The Induced pluripotent stem (iPS) cell technology was pioneered by Shinya Yamanaka and colleagues. They introduced four specific genes (Myc, Oct3/4, Sox2, and Klf4) as convertors of somatic cells into pluripotent stem cells (Takahashi and Yamanaka, 2006; Tanaka et al., 2020). These bioengineered stem cells act similarly to embryonic stem cells and have successfully been created and converted to glucose-responsive insulin-producing cells *in vitro* (Migliorini et al., 2021; Fantuzzi et al., 2022). Therefore, iPSCs are considered a promising source for the generation of a large number of β-cells from an autologous non-embryonic tissue origin (Liu G. et al., 2020).

iPSCs have been generated from donor’s skin, or blood cells and patient-specific cells. The iPSC technology holds promise in the field of regenerative medicine since iPSCs can propagate and give rise to different cell types such as neurons, muscle cells, pancreatic, or liver cells, etc (Mao et al., 2022; Hirschi et al., 2014). They represent a single source of cells that can be used to replace cells lost to damage or disease. Because these patient-specific cells are derived from the patients' own cells (Mohammedsaleh, 2022), there would be no need to give patients any immunosuppressive drugs after transplantation, as is necessary for pancreas and islet cell transplants today (Bellin and Dunn, 2020; Chen and Gunton, 2021). A variety of stem cells from different tissue sources including adult- and embryonic stem cells (ESCs) are competent in giving rise to iPSC.

The most common approach for iPSC generation is the introduction of genetic material into the recipient genome using viral vectors. However, this approach possesses a risk of oncogenesis and tumorogenesis (Shao and Wu, 2010; Beghini et al., 2020).

Melton et al. revealed that functional human stem-cell-derived β-cells can be directly generated from human iPSC *in vitro*. These cells function like native human β-cells both *in vitro* and *in vivo* after transplantation (Pagliuca et al., 2014; Lu et al., 2017). Stem cell-derived β-cells have been able to produce insulin similarly to adult mature β-cells in response to glucose concentrations *in vitro*. Interestingly, these cells are able to express human insulin in mice after transplantation in a glucose-responsive manner (Chen et al., 2009). Furthermore, transplantation of these cells reduced hyperglycemia in diabetic mice (Chen et al., 2009; Alipio et al., 2010). More recent studies from the same group indicated that hiPSC derived from T1D patients can give rise to functional β-like cells *in vitro*. These β-like cells have been responsive to some of the anti-diabetic pharmaceuticals that are known to accelerate insulin production (Bourgeois et al., 2021), and suggests that stem cell-induced-β-like cells from T1D patients can be used not only for the treatment of diabetes but also for personalized drug screening and studies around drug processing. Interestingly, protocols have been established to promote the efficiency of iPSC differentiation to functional and active islet cells (Shahjalal HM. et al., 2018; Bourgeois et al., 2021; Soejitno and Prayudi, 2011; Hebrok, 2012)^,^.

Recently a clinical study has suggested a strategy for using iPSCs to generate growth factor- and IL-10-secreting T regulatory cells (Tregs) able to block undesired immune targeting of the recipient upon transplantation in mice (Haque et al., 2019; Haque et al., 2016). Human iPSCs derived through reprogramming of human somatic cells (e.g., keratinocytes and fibroblasts) can be appropriate substitute candidates for human ESCs (Liu G. et al., 2020; Bilousova et al., 2011; Hewitt et al., 2011). In one study, researchers established a method for the production of iPSCs using adenoviruses^164,165,166.^ The trans-differentiation of stromal cells into mature insulin-producing pancreatic β-like cells was enhanced and β-cell markers such as PDX1, NKX6-1 were expressed that are known to promote β-cell maturation (Pan et al., 2019; Baeyens et al., 2018; Nasteska et al., 2019).

Viral vectors based on retroviruses have long been used for iPSC generation because of their promising efficacy (Hu, 2014; Vargas et al., 2016). These studies have made great progress in resolving the ethical and safety issues around iPSC therapy including their involvement in the treatment of diabetes (Volarevic et al., 2018). However, endocrine cells derived from iPSCs using differentiation protocols *in vitro* often appear to have heterogeneous properties and express genes and phenotype related to thier immaturity (Bourgeois et al., 2021; Legøy et al., 2020).

## 6 Trans-differentiation of other pancreatic cell types into β-cells

The conversion of fully committed pancreatic cells such as duct-, acinar-, and α-cells into functional β-cells is another strategy for β-cell mass replenishment.

### 6.1 α-to β-cell conversion

Using α-cells as a source for generating new β-cells has been the focus of many investigators in the past decade (Habener and Stanojevic, 2012; Lu et al., 2014; Wei et al., 2022; Dahiya et al., 2024).

Near total ablation of α-cells using diphtheria toxin has been shown to promote spontaneous α-to β-cell conversion in mice (Thorel et al., 2010). In mouse models, the expression of β-cell-specific markers, e.g., PDX1 and PAX4 and Maf-A, as well as impaired expression of Arx and Dnmt1 in α-cells lead to the trans-differentiation of α-cells to β cells (Zhong and Jiang, 2019; Hunter and Stein, 2017; Nishimura et al., 2015).

In 2009, Collombat et al. reported the trans-differentiation of glucagon-producing cells into functional β-cells upon overexpression of PAX4 (Collombat et al., 2009a; Collombat et al., 2009b). In a different set of experiments, they blocked the expression of aristaless related-homeobox (known as ARX) in α-cells (Miura et al., 1997; Marsh and Golden, 2013). Cumulatively, both studies demonstrated that the overexpression of PAX4 and/or blockade of ARX led to the generation of functional β-cells. The regenerated β-cells were also shown to be able to reverse diabetes in STZ-treated transgenic mouse models (Yin et al., 2006).

Further, studies reported that the absence of glucagon-producing cells ran an endocrine-specification setting in the duct epithelium progenitor cells upon re-expression of NGN3^+^ in these uncommitted cells (Prasadan et al., 2010; Magenheim et al., 2011). This alteration in the endocrine phenotype was also detected upon diphtheria toxin-mediated α-cell death (Habener and Stanojevic, 2012; Thorel et al., 2010). There are also several reports indicating the trans-differentiation from α-to β-cell under certain culture medium supplements, drugs and conditions *in vitro* (Yu and Xu, 2020; Xing et al., 2023; Sarnobat et al., 2023; Nihad et al., 2021). The question, however, still remains as to whether these regenerative aspects can take place in humans since most of the studies have been carried out in rodents.

Another important question is regarding the translation of these findings into potential pharmacological protocols and/or cell based-therapy strategies. In 2009, a study revealed that the expression of Pax4 was enough for α-cells to trans-differentiate into β-cells (Gittes, 2009). Also, lack of Pax4 expression leads to loss of β-cells identity while boosting α-cells quantity (Collombat et al., 2009b). The data clearly indicates the developmental relation of these two pancreatic hormone-releasing cell types and their close gene expression profile. One year later in 2010, Herrera and colleagues showed that reprogramming of α-cells towards β-cells can be initiated by β-cell ablation (Thorel et al., 2010). They conducted their experiments by developing a transgenic mouse and used lineage tracing method for α-cells while ablated β-cell thoroughly using diphtheria toxin receptor machinery. The findings indicated that a strategy to completely destroy β-cells promoted regeneration of β-cells that are mainly developed from residual α-cells. Most interestingly, not only did the cells start to express β-cell specific biomarkers such as Pdx1 and Nkx6.1, but they also started to secrete insulin. Here again, the similarity in the function and origin of these 2 cell types might be the reason for α-to β-cell trans-differentiation. What pushes the α-cells to convert into insulin-producing cells is still a matter of debate. One theory is that extreme, abnormal conditions such as lack of insulin signaling in the microenvironment is responsible for initiating a series of changes that ultimately activates the conversion system. There are reports indicating that α-cells possess the Pdx1 and Mafa genes in their DNA content, which are known to present actively in β-cells (Thorel et al., 2010). In a study aimed at investigating the potential of transcriptional reprogramming, pancreatic islets were exposed to histone methyltransferase inhibitor that eventually led to the expression of Pdx1 biomarker and insulin production in glucagon producing α-cells (Bramswig et al., 2013; Spaeth et al., 2016).

A study published by Furuyama et al. reported that they have isolated islet non-β-cells admix with α-cells and pancreatic polypeptide (PPY)-producing γ-cell population from non-diabetic or diabetic human donors (Furuyama et al., 2019). The cells then undergo lineage-tracing and reprogramming using the transcription factors PDX1 and MAFA to produce insulin in response to glucose. Upon cellular transplantation into diabetic mice, converted human α-cells reverse diabetes by producing insulin up to 6 months post-transplantation (Furuyama et al., 2019). This study indicated that insulin-producing α-cells could still express α-cell markers detected by deep transcriptomic and proteomic profiling characterization. These findings also provide insight into molecular mechanisms for the treatment of Diabetes (Furuyama et al., 2019).

In an attempt to investigate conversion strategies to regenerate insulin-producing cells, Li et al. published a paper with an approach to trans-differentiate glucagon-producing α-cells to insulin-producing β-like-cells (Li et al., 2017). It was already clear that the loss of regulatory transcription factor Arx is adequate to induce trans-differentiation of α-cells to functional insulin-producing-β-like cells (Li et al., 2017). Based on this knowledge, Li et al. identified the small molecule Artemisinin, an antimalarial medication, as a playmaker capable of repressing Arx. They showed that the mechanism of action of these molecules depends positively on GABAA receptor signaling (Li et al., 2017).

A head-to-head study published by Ben-Othman et al. (Ben-Othman et al., 2017) reported the identification of GABA as an inducer of α-to-β-like cell trans-differentiation *in vivo*. This trans-differentiation process induced mechanisms of α-cell replacement through mobilizing duct-lining precursor cells representing α-cell characteristics before conversion into β-like cells. This process was carried out solely through GABA exposure (Ben-Othman et al., 2017). The study also indicated that the newly generated β-like cells could reverse STZ-induced diabetes *in vivo* (Ben-Othman et al., 2017). This study underscored the treatment of transplanted human islets with GABA leading to a loss of α-cells and an enhancement in β-like cell counts and α-to-β-like cell trans-differentiation process also in humans. The last two studies both published in 2017 made a stir at the time. They indicated that α-cells can convert into functional insulin-producing β-like-cells via progenitor cells using the drug GABA.

However, ever since, there have been reports of a lack of reproducibility of these results. Several researchers, including Ackermann et al. (Ackermann et al., 2018), and Rohban, the author of this review, tried to reproduce the results indicated in these two studies and failed. As a result, these publications are currently considered to be dubious papers.

It should be again noted, however, that most of these experiments and related findings have been carried out in rodents, particularly mouse models. To date, the ‘extreme β-cell loss’ leading to α-to β-cell conversion cannot be recapitulated in T1D human patients due to the lack of suitable immunosuppressive and immunoregulatory strategies. The immunosuppressive and immunoregulatory role of adult stem cells in particular mesenchymal stromal cells have been revealed that it might be worth trying to see if these stromal cells can play a dual role in differentiating into insulin-producing cells while suppressing the immune system in T1D patients.

### 6.2 Pancreatic ductal cells (PDCs)

PDCs form around 35% of the human pancreatic cell population (Lemper et al., 2015). These cells can be isolated *in vitro*, and they bear trypsinization and other enzymatic dissociation and cell isolation procedures (Gmyr et al., 2001; Dorrell et al., 2008). These properties make PDCs good candidates for expansion experiments aiming to increase the cell mass required for transplantation. PDCs maintain their proliferation capacity *in vitro* including in 3D cell culture systems using collagen, matrigel, or agarose as a matrix. However, without manipulation, these cells undergo cell-cycle arrest rapidly (Jurczyk et al., 2014). In 2014, Jin and colleagues reported that PDCs develop cystic bodies when cultured *in vitro* that accelerate their proliferation capacity. They also acquire insulin expression after approximately 7 days *in vitro* (Jin et al., 2014).

The use of growth factors, e.g., EGF, hepatocyte growth factor (HGF), KGF, or nicotinamide to stimulate human PDC proliferation has been tested in several studies (Corritore et al., 2016; Bonner-Weir et al., 2000; Rescan et al., 2005; Hoesli et al., 2012). However, most of them failed to bypass the incidence of rapid senescence and restricted proliferation rate and growth. Mouse tissue, however, represented a different growth pattern, as reported by Oshima et al. (Oshima et al., 2007). They used purified CD133^+^/c-Met^+^ PDCs with HGF, EGF, nicotinamide, and dexamethasone substances as culture medium additives.

Most of the protocols that worked successfully for mouse PDC expansion did not show reproducibility in human cells. This could be due to telomere shortening in human cells, which limits the replication capacity and trigggers senescence. Contrary to human cells, telomere length faces minimal alteration during subculture in rodents (Bonner-Weir et al., 2010). In addition, PDCs alter their phenotypic characterization soon after enzymatic treatment *in vitro*. The loss of E-cadherin followed by mesenchymal biomarkers such as N-cadherin and Snail1 known as master regulators of epithelial-mesenchymal transition (EMT) occurs (Corritore et al., 2016; Bonner-Weir et al., 2010).

The expression of mesenchymal characteristics by PDCs has been initially detected in human exocrine cells *in vitro* (Fanjul et al., 2010). However, in several studies, this phenomenon was also observed in human primary islet cultures (Fanjul et al., 2010; Gershengorn et al., 2004; Ouziel-Yahalom et al., 2006). These human islet cells also showed the capability of redifferentiating into insulin-producing cells after a higher proliferation rate. The properties of these cells were further studied by other groups through experiments such as lineage tracing (Baeyens et al., 2018; Chen et al., 2019). In terms of these investigations, they were also able to produce β-like-cells with the potency to reduce blood glucose levels in NOD mice (Nir et al., 2007; Verhoeff et al., 2021). The EMT process can occur in human PDCs to stop the early senescence of cells within primary cultures *in vitro* (Corritore et al., 2016).

Using FACS-sorted CA19-9^+^ PDCs, it was revealed that the purified cells within the culture system were proliferating in the presence of endothelial growth media (Corritore et al., 2016). The cells were characterized and identified as human duct-derived cells (HDDCs) with a massive expansion ability (Corritore et al., 2016). Moreover, an E−to N-cadherin switch and low levels of CK19 and SOX9 expression was detected in HDDCs (Corritore et al., 2014). Another set of experiments revealed that incubation of CA19-9+ cells with TGFβ inhibitor A-83–01 blocked the normal characteristics of HDDCs, including the inability to clonally expand. in vitro (Corritore et al., 2014; Corritore et al., 2015). Culture conditions were designed in a way that mimicked pancreatic embryonic development (Guo T. and Hebrok M., 2009). In this regard, small molecules and growth factors were used to recapitulate the desired condition (Pavathuparambil Abdul Manaph et al., 2019; Kondo et al., 2017). After 2-week incubation time, HDDCs were finally prone to express typical β-cell features, including insulin production capability, albeit without glucose sensitivity properties (Cantley and Ashcroft, 2015). These findings promoted further works around the evaluation of HDDC diabetes-reversal potential in relevant animal models (Kumar et al., 2012; Adigbli et al., 2020).

In 2008, Inada and colleagues hypothesized that progenitor cells are duct epithelial cells that undergo regression to a less differentiated state post-replication and can form new endocrine and exocrine pancreas (Inada et al., 2008). To test their hypothesis on whether ductal cells can act as pancreatic progenitors postnatally and give rise to new islets, transgenic mice expressing human carbonic anhydrase II (CAII), a duct marker, were generated. The study demonstrated that CAII-expressing cells within the pancreas function as progenitor cells capable of forming new islets and acini after birth and upon injury (Inada et al., 2008). Despite some limitations in the report (CreER was not knocked into the CAII gene), the paper illustrates the existence of a “duct to β-cell” path by doing lineage tracing using CAII, (the duct marker). These cells can serve as sources for new islet regeneration for diabetes therapy (Inada et al., 2008).

In 2023, Doke et al. demonstrated the pioneering data on scRNA-seq analysis of human pancreas. Their study confirms a dynamic plasticity in the pancreatic tissue through an intermediate ducto-acinar stage leading to endocrine cell neogenesis (Doke et al., 2023). This dynamic plasticity also promotes trans-differentiation of non-β-cells into mature β-cells in the pancreas (Honzawa and Fujimoto, 2021).

### 6.3 Pancreatic acinar cells

The isolation of pancreatic islets gives rise to a large population of acinar cells that can be distinguished from all other pancreatic cells (Puri and Hebrok, 2010). Soon after the isolation of these cells, scientists explored their ability to convert into β‐cells both *in vitro* and *in vivo* (Lysy et al., 2013; Donga and Wu, 2018). They also found out that in an intermediate phase during acinar-to β‐like-cell conversion, a population of duct cells was generated (Basile et al., 2019). It was first in 1992 that Lemoine and colleagues revealed the ability of human acinar cells to convert into CK-19^+^ duct cells (Hall and Lemoine, 1992). In 2005 and later 2007, other studies conducted by Lardon et al. and Miyawaky et al. showed that rodent pancreatic exocrine cells can be converted into insulin‐expressing cells when treated with LIF and EGF (Okuno et al., 2007; Baeyens et al., 2005). The co-transplantation of these cells together with acinar cells into immune-deficient mouse models resulted in the trans-differentiation of acinar cells into endocrine cells. After isolation and upon purification, these cells were referred to as “non‐endocrine pancreatic epithelial cells” (Hao et al., 2006). They were able to go through endocrine differentiation and promote the survival of pancreatic progenitor population in the epithelium layer of the pancreas (Hao et al., 2006).

In 2008, a group of scientists used adenoviruses for transporting Ngn3‐, Pdx‐1‐, and MafA genes for reprogramming processes within acinar cells (Zhou et al., 2008). The outcome gave rise to reprogrammed acinar cells that were able to produce insulin and decrease blood glucose levels in diabetic animals (Okuno et al., 2007). In contrast, Aldibbiat and colleagues showed in the same year that the converted acinar cells lacked the ability to process pro-insulin in trans-differentiated pancreatic acinar cells. This is due to deregulated insulin secretion and aberrant secretory pathway (Alidibbiat et al., 2008).

In a study in 2014, scientists induced the expression of Ngn3, Pdx‐1, and MafA through elastase 2A which is known to be a specific inducer of acinar cells. These genes were then introduced into exocrine cells (Li et al., 2014; Kim and Lee, 2016). The results showed that these genes play a distinct role in the process of conversion within the exocrine cells. It was further reported that Ngn3 and MafA expression mainly lead to trans-differentiation: Ngn3 gives rise to the pancreatic three islet endocrine cell types known as α‐, β‐ and δ‐cells. On the other hand, MafA showed to suppress the acinar and δ‐cell phenotypic properties while leading them to express more of α‐ and β‐cell properties. Pdx-1 has been also shown to play a role in the suppression of δ‐cell differentiation while directing them through β‐cell differentiation (Gannon et al., 2008). This study reported that the three main cell types in the pancreatic islets have been originated from the conversion of the acinar cells (Li et al., 2014). In another work, scientists were able to induce differentiation in murine acinar cells using substances such as nicotinamide and EGF (Baeyens et al., 2014; Wedeken et al., 2017). Using the lectin-labeling method for the acinar cells, they demonstrated that the regenerated β‐like-cells were of acinar cell-origin (Kim and Lee, 2016). Others such as Baeyens and colleagues in 2006 also reported that during β‐like-cell generation, kinase-associated signaling pathways such as JAK/STAT liaise with Ngn3 expression to promote β‐cell neogenesis (Baeyens et al., 2006). They highlighted that blocking Ngn3 expression by impairing EGF and LIF signaling pathways, as well as inhibiting EGFR, JAK2, and STAT3, led to a significant reduction in the formation of new insulin producing β‐like-cells in the pancreas. Normally, EGF and LIF signaling, along with molecular factors, promote the re-expression of the Ngn3 molecule in adult pancreatic cells through the JAK/STAT pathway. Conversely, inhibiting JAK/STAT led to a decrease in Ngn3 expression. This process is similar to neurogenesis, where EGF enhances the responsiveness of LIF signaling via the STAT3 cascade. Overall, the study demonstrated that EGF and LIF signaling through the JAK/STAT pathway promotes the re-expression of Ngn3 in pancreatic cells (Baeyens et al., 2006).

The researchers also determined that acinar-to β‐like-cell conversion takes place in the acinar cells upon MAPK overexpression (Lemper et al., 2015; Kim and Lee, 2016). Here gene therapy comes in handy for delivering the genes to the acinar cells through lentiviruses and/or microRNA manipulation, which resulted in overexpression of β‐cell-specific biomarkers, e.g., insulin and Pdx‐1 in the cells (Corritore et al., 2016; Williams et al., 2020). It is interesting to know that the acinar-to β‐cell conversion worked more effectively *in vitro*, e.g., in 3D culture methods (Yu and Xu, 2020; Baeyens et al., 2018; Hebrok, 2012). A lineage-tracing method using adenovirus recombinase elastase 2A promoter confirmed the origin of Ngn3‐ and insulin‐producing cells to be human acinar cells (Kim and Lee, 2016). The finding indicated the ability of human exocrine cells to convert into β‐like insulin‐expressing cells. Not only was it a groundbreaking scientific finding, but it also leads to a novel therapeutic and regenerative strategy in which plenty of exocrine cells can be obtained and used for regenerative therapy purposes. All in all, it is now known that acinar cells have the potential to trans-differentiate into β‐like insulin‐producing cells both *in vitro* and *in vivo*. Hence they serve as a promising source of cells that can be used in the treatment and even cure of diabetes.

Very recently in 2024, a study conducted by Esni and collegues demonstrated that preventing focal adhesion kinase activity leads acinar cells to convert into funtional, glucose responsive β-like cells (Dahiya et al., 2024), a significant step forward through diabetes cure.

## 7 Comparison of effectiveness of various insulin-producing cell generation protocols

Comparing the effectiveness of various insulin-producing β‐like cell differentiation protocols and regenerative strategies sheds light into potential opportunities and challenges in advancing the diabetes treatment (Gabr et al., 2017; Boyd et al., 2008). Several approaches have been developed to differentiate stem cells into insulin-producing cells, each with its unique effectiveness, advantages and limitations (Enderami et al., 2017; Nemati et al., 2021; Liew, 2010; Schroeder et al., 2006; Seissler and Schott, 2008; Shi, 2010). These approaches involve.

### 7.1 Pancreatic progenitor cells protocol

This protocol involves differentiating stem cells into pancreatic progenitor cells, which can further mature into β‐ cells that produce insulin. While this method has shown promising results in preclinical studies, it requires precise control of developmental signaling pathways to ensure the efficient generation of functional β‐cells (Hogrebe et al., 2021; Shim et al., 2007; Shi, 2010).

### 7.2 Directed differentiation protocol

In this approach, stem cells are exposed to a defined series of growth factors and signaling molecules to mimic the natural developmental process of the pancreas. This method has the advantage of producing insulin-producing cells that closely resemble native β‐cells in both structure and function. However, the protocol can be complex and time-consuming, and requiring optimization for each cell line used (Shim et al., 2007; Zhang et al., 2009; Trounson, 2006).

### 7.3 Trans-differentiation protocol

Trans-differentiation involves directly converting non- β‐cells, such as liver cells, into insulin-producing cells. This method offers the advantage of bypassing the pluripotent stem cell stage, and reducing the risk of tumour formation. However, the efficiency of trans-differentiation can vary among cell types and may require genetic manipulation to enhance the conversion process (Yang et al., 2002; Okura et al., 2009; Oh et al., 2004).

### 7.4 3D culture system protocol

Utilizing 3D culture systems can enhance the differentiation efficiency of stem cells into insulin-producing cells by providing a more physiologically relevant microenvironment. The spatial organization within 3D cultures can promote cell-cell interactions and signaling pathways crucial for beta cell maturation. However, optimizing 3D culture conditions for scalability and reproducibility remains a challenge (Khorsandi et al., 2015; Mendoza et al., 2018; Liang et al., 2023).

While each insulin-producing cell differentiation protocol posesses a certain level of effectiveness and has its strengths and limitations, a combination of these approaches may hold the key to maximizing efficiency and functionality. Future research should focus on refining existing protocols, identifying novel molecular targets, and developing standardized methodologies for generating reliable sources of insulin-producing cells for diabetes therapy (Gabr et al., 2017; Boyd et al., 2008; Enderami et al., 2017; Nemati et al., 2021; Shi, 2010).

## 8 Long-term stability and functionality of generated insulin-producing β‐cells *in vivo*

Insulin-producing β‐cells replacement therapy holds great promise for patients with diabetes. However, its success lies in the long-term stability and functionality of generated β‐cells *in vivo* (Benthuysen et al., 2016; Ramzy et al., 2023). This depends on several factors.

### 8.1 Cell source and differentiation

The choice of cell source for generating insulin-producing β‐cells signifacantly influences their long-term stability and functionality post-transplantation (Lock and Tzanakakis, 2007; Jun Hee-Sook and Park Eun-Young, 2009)^,^. Differentiation protocols, stem cell sources, and genetic manipulation and editing techniques all play crucial roles in determining the quality and maturity of β‐cells (Guo Tingxia and Hebrok Matthias, 2009; Melton, 2021). Recent advances in generating functional β‐cells from pluripotent stem cells and other sources provide optimism for enhancing long-term cell survival (Lock and Tzanakakis, 2007; Jun Hee-Sook and Park Eun-Young, 2009; Guo Tingxia and Hebrok Matthias, 2009; Melton, 2021).

### 8.2 Transplantation techniques

Optimal transplantation techniques are essential for promoting the engraftment and survival of generated β‐cells *in vivo*. Site of transplantation, encapsulation technologies, and vascularization strategies all impact the long-term functionality of transplanted β‐cells (Rohban Rokhsareh et al., 2016; Farina et al., 2019; Pellegrini et al., 2016; Shahjalal HussainMd et al., 2018; Köllmer et al., 2016). Novel approaches such as bio-engineered scaffolds offer innovative solutions to improve the integration and longevity of β‐cell transplants (Gazia et al., 2019; Malik and Dhasmana).

### 8.3 Immune responses and rejection

Immune responses against transplanted β‐cells possess a significant challenge to their long-term stability and functionality (Tahbaz and Yoshihara, 2021; Caldara et al., 2023; Shi et al., 2022). Strategies to modulate the immune system, including immunosuppressive drugs, immune-privileged sites, and cell encapsulation, must be carefully considered to prevent rejection and ensure sustained cell survival (Tahbaz and Yoshihara, 2021; Caldara et al., 2023; Köllmer et al., 2016; Johannesson et al., 2015).

### 8.4 Cell survival and functionality

Promoting the long-term survival and functionality of transplanted β‐cells requires a multi-faceted approach. Factors such as oxygen tension, nutrient supply, and inflammatory environment all impact the viability and insulin-secreting capacity of β‐cells (Yitayew et al., 2024; Shi et al., 2022; Lazard et al., 2012; Ghasemi et al., 2021). Novel insights into enhancing cell survival through tissue engineering, gene editing, and co-culture systems offer exciting possibilities for improving the long-term outcomes of β‐cell therapy (Abadpour et al., 2021; Ghezelayagh et al., 2021; Gamble et al., 2018; Orlando et al., 2014).

## 9 Cell protection: a side-approach

Although this approach is not about the regeneration of β-cells, it is relevant for any therapeutic strategy against diabetes. Researchers have identified new protein components that may be involved in protecting islets and in particular insulin-producing-cells from being attacked by the immune system. The immune interactions within the pancreatic islet play a crucial role in the incidence of T1D and the survival of transplanted-cells aimed at treating the disease (Cobo-Vuilleumier and Gauthier, 2020). Strategies for protecting cells against autoimmune attack would enable affected patients to delay, reverse, or prevent the onset of T1D (Roep et al., 2021). Studies conducted in diabetic animal models have revealed that pancreatic delivery of a synthetic protein can stop diabetes incidence in relevant drug-induced diabetic mice (King, 2012; Nakatsuma et al., 2016).

It is believed that an efficient strategy for treating/curing T1D should be able to provide/restore the functionality of β-cells. But it is also believed restoring endogenous‘ β-cell mass without having a combinatorial strategy to protect them from immune attack will just be half-road towards T1D treatment. In 2018 Gittes and colleagues used a gene therapy approach by infusing Pdx1^+^ and MafA^+^ adenovirus in the pancreatic duct (Xiao et al., 2018). Their aim was to reprogram ɑ-cells into functional β-cells using β-cell-toxin-induced diabetic mice and non-obese diabetic (NOD) mouse models. The results indicated that upon this intervention, the blood glucose level in both rodent models was normalized and persisted for 4 months before the reoccurrence of autoimmune diabetes. Moreover, using this gene therapy strategy, the ɑ-to β-cell trans-differentiation was also promoted in human pancreatic islets that have been exposed to viral transduction. It has been presumably due to the immunosuppressive approach of the strategy. The purpose was not only to push the cells to produce insulin, but also to delay immune reaction leading to the destruction of newly established β-cells.

## 10 Regenerative strategies for β-cell regeneration: From bench to bedside

## 11 Regulatory challenges and ethical considerations

The use of stem cells and gene editing technologies presents a myriad of regulatory challenges and ethical considerations that must be carefully navigated. From a regulatory standpoint, one of the primary concerns is ensuring that research and applications involving these technologies adhere to established guidelines and safety measures (Rossi et al., 2009). Regulatory parties must monitor the use of stem cells and gene editing to prevent potential misuse, unethical practices, and unforeseen consequences (Rossi et al., 2009; Nordberg et al., 2020).

Ethical considerations surrounding these technologies are complex and multifaceted. Concerns include the potential for exploitation of vulnerable populations, such as through the commercialization of stem cell therapies (Kandi and Vadakedath, 2022; Lo and Parham, 2009; Karagyaur et al., 2019). There are also ethical issues related to the creation, usage, and destruction of embryos for research purposes, as well as the implications of editing the human genome content and passing genetic modifications onto future generations (Kandi and Vadakedath, 2022; Lo and Parham, 2009; Borge and Evers, 2003; Karagyaur et al., 2019).

Furthermore, issues of consent, privacy, and equity must be carefully addressed in the context of stem cell and gene editing research. Ensuring that individuals understand the risks and benefits of these technologies, as well as protecting their rights and autonomy, is crucial for upholding ethical standards (Lo and Parham, 2009; Palazzani, 2019).

The regulatory challenges and ethical considerations on the use of stem cells and gene editing technologies underscore the need for thoughtful oversight, transparent and ongoing communication between researchers, policymakers, and the public to maintain ethical and regulatory standards (Lo and Parham, 2009; Kohn et al., 2016; Karagyaur et al., 2019).

## 12 Translational aspects towards human application

Translational findings on the generation of insulin-producing β-cells are of utmost importance for bringing the animal and rodent model findings to human based-clinical trials for diabetes treatment (Basile et al., 2022; Jiang et al., 2018). One key concept elucidated in recent studies is the importance of understanding the signaling pathways and molecular mechanisms involved in the differentiation of β-like-cells from different precursor cell types (Bernal-Mizrachi et al., 2014; Stewart et al., 2015; Mullen and Wrana, 2017; Mossahebi-Mohammadi et al., 2020; Huang et al., 2020). Through uncovering the cellular processes and signaling pathways that conduct this differentiation, is it possible to work towards developing novel strategies to generate functional β-cells for diabetes therapy in a clinical trial setting (Jiang et al., 2018).

Original insights have emerged from studies focusing on the role of key transcription factors, such as PDX1, NKX6.1, and MAFA, in driving β-cells differentiation (Pan et al., 2019; Baeyens et al., 2018; Memon et al., 2018; Nasteska et al., 2019). Manipulating the expression of these transcription factors through genetic or pharmacological approaches has shown promise in enhancing the efficiency of insulin-producing β-like-cells generation (Jiang et al., 2018; Soria et al., 2015). Additionally, researchers are exploring the use of gene editing technologies, such as CRISPR/Cas9, to precisely modulate gene expression and improve the functionality of generated insulin-producing β-like-cells, to be used in human settings through clinical trials (Hu et al., 2020).

Moreover, the field is advancing with the incorporation of innovative biomaterials and microenvironmental cues to mimic the native pancreatic niche and enhance the maturation and function of β-cells (Patel et al., 2022). Techniques such as 3D bio-printing and organoid culture systems are being explored to create more physiologically relevant platforms for generating insulin-producing cells (Kumar et al., 2019; Salg et al., 2019).

There are, however, challenges that must be sorted out or minimized to ensure successful application in clinical settings (Eizirik et al., 2020). One key challenge lies in the inherent differences between animal and human biology, which can impact the behavior and functionality of β-like-cells (Nair et al., 2020). For instance, variations in cell surface markers, hormonal regulation, and immune responses may lead to difference in the way β-like-cells function in humans compared to animal and/or rodent models (Benthuysen et al., 2016). As a result, findings from animal studies may not always directly translate to human setting.

Another significant challenge is the complexity of human diseases such as diabetes, which can manifest differently in individuals compared to laboratory animal models (Eizirik et al., 2020; Salinno et al., 2019; Pound and Ritskes-Hoitinga, 2018; Denayer et al., 2014; McGonigle and Ruggeri, 2014). Factors like genetic environmental factors, and disease progression can all contribute to variations in how insulin producing β-like-cells respond to different treatments or interventions (Salinno et al., 2019; Denayer et al., 2014). This highlights the importance of considering the heterogeneity of human populations when translating research findings from animal models to human contexts (Pound and Ritskes-Hoitinga, 2018; Denayer et al., 2014; McGonigle and Ruggeri, 2014).

Furthermore, practical considerations such as reproducibility and safety also bring challenges in translating findings on insulin producing β-like-cells to human applications (Basile et al., 2022). Scaling up production of these cells for clinical use, ensuring consistent quality and functionality, and addressing potential safety concerns are critical aspects that must be carefully evaluated and optimized to enable successful translation to human contexts (de Almeida Fuzeta et al., 2020; Robb et al., 2019; Fernández-Santos et al., 2022).

## 13 Opportunities, challenges, limitations and risks

The field of advanced medicine is rapidly growing. In contrast, stem cell therapy as one remarkable category remains purely theoretical or limited to animal experiments that are not always translatable to human therapeutic settings (Sng and Thomas, 2012).

As of yet, the potential playmakers in the process of β-cell regeneration involve embryonic-, adult- and hematopoietic stem cells, and pancreatic progenitor cells, other pancreatic committed cells such as acinar-, duct- and ɑ-cells (through conversion into functional β-like-cells), iPSC, and small druggable molecules with the capacity to direct stem cell fate toward insulin-producing β-like cell development.

In this regard, cellular conversion has gained momentum in generating insulin-producing, glucose-sensing, functional β-like-cells with a trans-differentiation approach that may hold the key to finding a cure for T1D and T2D.

Using mesenchymal stromal cells in β-cell regeneration strategies against T1D might have significant dual benefits. Not only do these cells have the capacity to regenerate insulin-producing cells, but they can also regulate immunity by their immunosuppressive properties crucial in the treatment of autoimmune T1D.

The most significant limitations of human stem cell therapies are related to 1) the number of patients participating in clinical trials and 2) the duration of clinical studies. These trials often involve a small group of participants and are performed over a short period. The timing in clinical trials is of utmost importance as the limited project duration might hamper the precise evaluation of risks, such as carcinogenesis: a potential side-effect of stem cell therapy. As a result, the trial outcome may not be reliable for long-term safety and efficacy evaluations.

Besides, the lack of personalized perspective in designing a therapy for a patient results in using generic therapies for a heterogeneous population of patients regardless of individual genetic and epigenetic factors.

Another limitation is that stem cell therapeutic interventions often focus on reducing disease symptoms rather than “curing” the disease altogether.

This is due to a biased mindset trying to treat only a single disease symptom but also due to the inability to correlate different aspects of the disease, or similar pathways that affect and control a series of disease symptoms.

To overcome these issues, combinational therapy comes in handy to treat various disease symptoms by a combination of different methods such as stem cell transplantation, gene therapy, and pharmacology for targeting multiple aspects of disease simultaneously.

Another limitation is due to a narrow understanding of the intracellular molecular pathways that regulate stem cell differentiation, and new methods to manipulate these pathways for inducing epigenetic modifications of desire.

Finally, an incomplete understanding of stem cell signaling and cellular communication hampers combating potential pitfalls such as immune rejection.

Potential risks are also related to ethical issues around stem cell therapy and notably using embryonic stem cells in designing clinical therapy mile.

Although many pioneering, groundbreaking, and game-changing discoveries have changed our understanding of stem cell function in cell therapy, further studies on stem cells and cell- and gene therapy are still required before a panacea with maximum therapeutic and cure potential for diabetes can be achieved (Shao and Wu, 2010; Beghini et al., 2020; Attwood and Edel, 2019; Ortuño-Costela et al., 2019; Yi et al., 2011; Nakanishi and Otsu, 2012; Park et al., 2016).
